# Validation of Novel Reference Genes for Reverse Transcription Quantitative Real-Time PCR in Drought-Stressed Sugarcane

**DOI:** 10.1155/2014/357052

**Published:** 2014-06-02

**Authors:** Roberta Lane de Oliveira Silva, Manassés Daniel Silva, José Ribamar Costa Ferreira Neto, Claudia Huerta de Nardi, Sabrina Moutinho Chabregas, William Lee Burnquist, Günter Kahl, Ana Maria Benko-Iseppon, Ederson Akio Kido

**Affiliations:** ^1^Federal University of Pernambuco (UFPE/CCB/Genética), 50670-420 Recife, PE, Brazil; ^2^ABC Medical School, 09060-650 São Paulo, SP, Brazil; ^3^Sugarcane Technology Center (CTC), 13400-970 Piracicaba, SP, Brazil; ^4^Institute of Molecular Biosciences, Goethe University Frankfurt am Main, 60438 Frankfurt am Main, Germany

## Abstract

One of the most challenging aspects of RT-qPCR data analysis is the identification of reliable reference genes. Ideally, they should be neither induced nor repressed under different experimental conditions. To date, few reference genes have been adequately studied for sugarcane (*Saccharum* spp.) using statistical approaches. In this work, six candidate genes (**α**TUB, GAPDH, H1, SAMDC, UBQ, and 25S rRNA) were tested for gene expression normalization of sugarcane root tissues from drought-tolerant and -sensitive accessions after continuous dehydration (24 h). By undergoing different approaches (GeNorm, NormFinder, and BestKeeper), it was shown that most of them could be used in combinations for normalization purposes, with the exception of SAMDC. Nevertheless three of them (H1, **α**TUB, and GAPDH) were considered the most reliable reference genes. Their suitability as reference genes validated the expression profiles of two targets (AS and PFP**α**1), related to SuperSAGE unitags, in agreement with results revealed by previous *in silico* analysis. The other two sugarcane unitags (ACC oxidase and PIP1-1), after salt stress (100 mM NaCl), presented their expressions validated in the same way. In conclusion, these reference genes will be useful for dissecting gene expression in sugarcane roots under abiotic stress, especially in transcriptomic studies using SuperSAGE or RNAseq approaches.

## 1. Introduction

Sugarcane (*Saccharum *spp.) is a major crop with vegetative propagation and capacity to accumulate high levels of sucrose in the culms [1–3]. World production of this crop in 2011 resulted in about two billion tons of raw material, which corresponded to a gross income of more than US$ 52 billion [4]. Despite the economic importance of sugarcane, the knowledge of relevant genetic mechanisms remains challenging, due to the fact that this crop presents one of the largest and most intricate genomes of the plant kingdom, with diploid numbers ranging from 100 to 130 chromosomes, indicating a high ploidy level, as well as regular aneuploidy events [5–7]. Because of this complexity, the use of molecular tools represents an attractive approach to the improvement of sugarcane breeding programs. Moreover, transcriptomic studies have been prioritized, allowing identification of candidate genes involved in developmental processes and plant responses to environmental cues, which have eventually led to the discovery of functional molecular markers [8]. Reverse transcription quantitative real-time PCR (RT-qPCR) is based on a high specific polymerase chain reaction associated with sensitive fluorescence, allowing the detection of variations in gene expression, including discreetly transcribed genes [9, 10]. This technology has been used as a diagnostic tool for identification of plant pathogens, transgene expression [9], and human diseases [11, 12] and confirmation of transcriptional profiles generated by different methodologies, such as EST libraries [13], Microarray [14], HT-SuperSAGE [15], and RNAseq [16]. The reliability of RT-qPCR data based on relative quantification is depending upon comparative transcription of target genes to stable reference genes [17, 18]. The use of reference genes that undergo changes in transcription across experimental groups can dramatically alter the conclusions on targeted gene expression [18]. In order to test for normalization of reference gene expression, several statistical algorithms, such as GeNorm [17], NormFinder [19], and BestKeeper [20], have recently been developed. Despite the importance of proper reference gene selection for reliable and accurate RT-qPCR assays, most reports involving sugarcane have not described, or compared, methods in order to determine the efficiency of reference genes [21–23], suggesting arbitrary criteria for this selection. To our knowledge, only one systematic study aimed to assess, standardize, and validate reference genes (GAPDH, *β*-tubulin, *β*-actin, and 25S rRNA) for tissue and genotype-specific gene expression analysis in sugarcane [24]. Additionally, this kind of study has not been carried out before under stress conditions which often alter the behavior of some genes. Thus, a rigorous selection of reference genes for expression profiling validation in sugarcane under biotic or abiotic stress was yet to be described. In the present work, screening and validation of new reliable reference genes for expression analysis in sugarcane roots were carried out. Besides, additional resources for target validation were evaluated, especially considering comprehensive transcription profiling, like those provided by HT-SuperSAGE [25], in sugarcane, revealing hundreds of candidate genes responsive against drought stress, requiring subsequent validation.

## 2. Materials and Methods

### 2.1. Plant Material and Treatments

Sugarcane drought assay: accessions were previously evaluated in a glasshouse trial conducted by the Center for Sugarcane Technology (CTC) in Piracicaba, Brazil (22° 41′ S; 47° 33′ W), aiming to identify drought-tolerant and drought-sensitive ones. Four accessions were selected as drought-tolerant (CTC6, CTC15, SP83-2847, and SP83-5073) and another four as drought-sensitive (CTC9, CTC13, SP90-3414, and SP90-1638). Some of them were previously reported as tolerant and sensitive to drought based on chlorophyll and water content measurements together with field observations [28]. Briefly, for the drought stress assay, plants of each accession were grown under glasshouse conditions (30.2 ± 5.7°C (maximum), 16.8 ± 1.9°C (average), and 9.3 ± 3.0°C (minimum) and 71.5 ± 5.1% (average) relative humidity under natural photoperiod) in 40 L pods in a randomized experimental design (comprising six repetitions) under daily irrigation (4 L·h^−1^) until reaching three months of age. Plants were submitted to drought conditions by continuous dehydration caused by the interruption of irrigation during 24 hours. Roots of both, stressed and unstressed plants, were collected, immediately frozen in liquid nitrogen, and stored at −80°C until total RNA extraction. On the other hand, the salinity stress assay was performed as follows: acclimated plants of the RB931011 clone* in vitro* cultivated (referred to as salt tolerant by the Brazilian RIDESA program of sugarcane breeding) were grown in a greenhouse (UFPE, Recife, PE, Brazil; 8° 04′S, 34° 55′W) in pots containing washed sand (washed) and they were watered daily with Hoagland solution, throughout three months. Later, NaCl (100 mM) was added to the nutritive solution as the salt stress. Roots from both stressed and nonstressed (negative control) plants were collected after stress induction (30 and 90 min) and immediately frozen in liquid nitrogen until a total RNA extraction was performed.

### 2.2. Total RNA Isolation, Purification, cDNA Synthesis, and HT-SuperSAGE Libraries

Total RNA was extracted with RNeasy Mini kit (Qiagen) according to the manufacturer's instructions, treated with DNAse (Qiagen), and purified with RNeasy Mini kit (Qiagen). RNA samples were quantified using Quant-iT RNA assay kit (Invitrogen) with the Qubit fluorometer (Invitrogen). RNA integrity was verified in 1.5% agarose gel electrophoresis with blue-green loading dye (LGC Biotechnology) staining. The purified RNA (1 *μ*g) of each sugarcane accession was reverse-transcribed using QuantiTect Reverse Transcription Kit (Qiagen) according to the manufacturer's instructions and resuspended in a final volume of 20 *μ*L. The cDNA synthesis reaction was incubated at 42°C for 2 minutes (genomic DNA digestion), 42°C for 15 minutes (reverse transcription), and 95°C for 3 minutes (enzyme inactivation) and stored at −20°C. The procedures for HT-SuperSAGE library generation followed Matsumura et al. [29], including the attachment of library-specific adaptors carried out by GenXPro GmbH (Frankfurt, Germany), allowing the identification of library-specific reads after SOLEXA sequencing. Concerning the drought stress, four libraries were generated as described by Kido et al. [25]: the bulk of tolerant accessions under stress and the respective negative control and the bulk of sensitive accessions after stress and the respective negative control. The bulks were composed by equimolar amounts of poly-A^+^ mRNA from all accessions comprising the respective library. In relation to the salt stress, equimolar amounts of total RNA from each sample/time were assembled to compose the two bulks used to generate the SuperSAGE libraries (stressed and control).

### 2.3. Primer Design, Amplification Efficiency, and RT-qPCR Analysis

Sugarcane ESTs (Table 1) from dbEST database (http://www.ncbi.nlm.nih.gov/nucest), related to independent pathways as an attempt to minimize the effects of coregulation, were used for primers design with the Primer 3 software [30] with minor modifications: the amplicon length range was set to 70–200 bp, the melting temperatures were between 40°C (minimum), 50°C (optimum), and 60°C (maximum), and the CG content ranged from 45 to 55% (optimum of 50%). In relation to H1 and SAMDC, the primers sequences were obtained from the literature [23]. All primer pairs were synthesized by Bioneer Corporation (South Korea) and some details of these primers are given in Table 1. An initial standard PCR was performed with the potential reference genes, using the sugarcane cDNA samples (bulk of tolerant and sensitive accessions), in order to investigate the PCR products. Amplicons were analyzed on 1.5% agarose gel electrophoresis followed by blue-green loading dye staining (LGC Biotechnology). Additionally, a dissociation curve analysis was made, in RT-qPCR assay, to confirm the specificity of the amplification by the candidate genes. Calibration curves using a dilution series of the cDNA pool (1, 10^−1^, 10^−2^, 10^−3^, and 10^−4^) were made to calculate the PCR amplification efficiencies (*E* = 10^−1/slope^) [27] for each quantified candidate gene, their respective correlation coefficients (*R*), and *y* interceptors. The RT-qPCR amplifications were performed on LineGene 9660 model FQD-96A (Bioer), using SYBR Green detection. Each reaction mixture comprised 1 *μ*L of template cDNA (diluted 5-fold), 5 *μ*L of HotStart-IT SYBR Green qPCR Master Mix 2x (USB), 0.05 *μ*L of ROX (normalization dye), 1.95 *μ*L of ultrapure water, and 1 *μ*L of each primer (0.05 *μ*M), forming a final volume of 10 *μ*L. Three biological and three technical replicates were used in each run for RT-qPCR analysis, and a no template control (NTC) was also included. The reactions were subjected to an initial denaturation step of 95°C for 2 min, followed by 40 cycles at 95°C for 15 s, 58°C for 30 s, and 72°C for 30 s in 96-well reaction plates. The dissociation curves were analyzed at 65–95°C for 20 min after 40 cycles. The baseline and quantification cycle (Cq) were determined using the LineGene 9600 software (version 1.1.10).

### 2.4. Data Analysis

The potential reference genes were ranked, and the number of candidate genes required for an optimal normalization was indicated according to their gene expression stability using sugarcane cDNA samples, after being analyzed by the GeNorm (version 3.5) [17], NormFinder (version 0.953) [19], and BestKeeper (version 1) [20] software. The GeNorm and NormFinder input data were based on relative quantities applying the ΔΔCq method [31]. The GeNorm software determines the reference gene stability measurement (*M*) as the average pairwise variation of each reference gene with all the other reference genes and enables the elimination of the least stable gene and the recalculation of the *M* values, resulting in the ranking of the most stable genes. The average expression stability value (*M*-value) was a parameter for quantification of stable reference gene candidates, in which a low* M*-value indicated a more stable expression [17]. The NormFinder tool was applied to identify and rank the most suitable genes for RT-qPCR normalization from the set of candidates, considering intragroup and intergroup variations, in a model-based approach of mixed linear effect modeling [19]. The BestKeeper software, using raw Cq values as input, was applied to identify the most stable expressed genes by a Pearson correlation coefficient (geometric mean of Cq values of candidate genes), calculating the standard deviation (SD) of Cq values among the entire data set. The relative gene expression levels (based on the relative quantities after the ΔΔCq method) were evaluated with the REST^©^ tool (Relative Expression Software Tool, version 2.0.13), which bases its performance on pairwise comparisons using randomization and bootstrapping techniques (Pairwise Fixed Reallocation Randomization Test^©^) [32]. The normalization of the RT-qPCR was performed by taking the geometric averages of the combined reference genes, using the negative control to normalize this relative expression, and testing the hypothesis of significant differences between the control and treatment. With the input of multiple target and reference genes and based on the normalized values of the target genes, the software indicates the direction of the difference between the groups, as well as their *P* value. Also, the MIQE guidelines (The Minimum Information for Publication of Quantitative Real-Time PCR Experiments) [26] were followed in order to increase transparency and reliability of the results obtained.

## 3. Results 

### 3.1. RNA Integrity, Specificity, and Efficiency Amplifications

All the reference candidate genes (*α*TUB, GAPDH, H1, SAMDC, UBQ, and 25S rRNA) amplified the cDNAs generated from the RNAs samples (Supplemental Figure 1(a) in Supplementary Material available online at http://dx.doi.org/10.1155/2014/357052) using the proposed primers. Based on standard PCR amplifications, only a single product was observed with a specific primer pair (Supplemental Figure 1(b)) and these results were supported by the dissociation curve analysis (Supplemental Figure 1(c)). Based on the standard curves using a serial dilution of the cDNA pool (Supplemental Figure 2), the real-time PCR amplification efficiency (*E*), considering the selected six candidate genes, ranged from 98.34% to 100.89%, with correlation coefficients (*R*) varying from 0.984 to 0.999, while the slopes ranged from −3.50 to −3.21 (Table 2). Considering the efficiency of 100%, the value of the expected slope should be −3.32, while slopes ranging from −3.10 to −3.58 would represent efficiency comprising 90% to 110%, thereby characterizing acceptable reactions. These parameters derived from the RT-qPCR analysis, and others in accordance with the MIQE Guidelines, are shown in Tables 2 and S2. The results showed favorable conditions for amplification, efficiency in successive dilutions, and acceptable variations in gene expression across samples, representing potential for choosing a suitable reference gene. Thus, in gene expression studies, fluctuations due to pipetting errors, variations in the quantification of samples, or the concentration of reagents could be normalized with the aid of these suitable reference genes [33].

### 3.2. Gene Expression Stability of the Reference Gene Candidates

The six candidate genes selected for normalization (*α*TUB, GAPDH, H1, SAMDC, UBQ, and 25S rRNA) in RT-qPCR tests showed Cq values ranging from 13.06 to 28.00 (Supplemental Table S1). Most of the candidate genes presented Cq values with slight variations (below one cycle), except UBQ and SAMDC. Based on these values, 25S rRNA was the most abundantly transcribed gene (average Cq = 14.00), while *α*TUB was the least abundant (average Cq = 27.48). These data, in order to assess the gene expression stability of the reference gene candidates, were used in GeNorm [17], NormFinder [19], and BestKeeper [20] analysis.

#### 3.2.1. GeNorm Analysis

**Figure 1:**
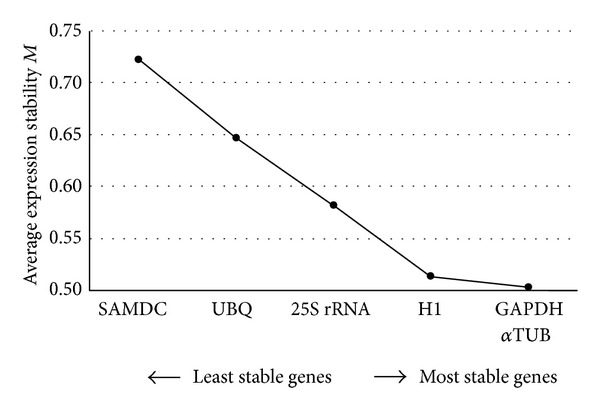
Average gene expression stability values (*M*) of six sugarcane potential reference genes (*α*TUB: alpha-tubulin; GAPDH: glyceraldehyde 3 phosphate dehydrogenase; H1: histone H1; SAMDC: S-adenosylmethionine decarboxylase; UBQ: ubiquitin; 25S rRNA: 25S ribosomal RNA) based on the GeNorm analysis [17].

**Figure 2:**
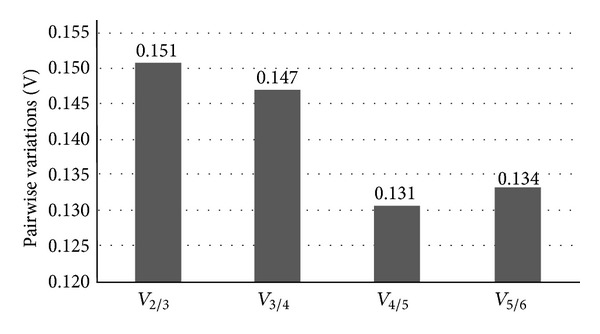
Pairwise variation (*V*) analysis for six potential reference genes of sugarcane (*α*-tubulin, glyceraldehyde 3 phosphate dehydrogenase, histone H1, S-adenosylmethionine decarboxylase, ubiquitin, and 25S rRNA) based on the GeNorm analysis [17]. The addition to the two most stable genes (*α*TUB and GAPDH) of a random third gene (*V*_2/3_ = 0.15), a fourth gene (*V*_3/4_ = 0.14), or even more (*V*_4/5_ and *V*_5/64_) still exhibited desirable values (basically below than 0.15).

Considering the average expression stability values (*M*-value), *α*TUB (*M* = 0.61), GAPDH (*M* = 0.62), and histone H1 (*M* = 0.63) were the most stable genes while SAMDC represented the most variable (*M* = 0.87) gene. However, all of them showed an expressive high stability with* M*-values below 1 (Figure 1), suggesting that all the six candidates may be adequate for normalizing gene expression data under the conditions used in the present work. Besides, based on the pairwise variation (*V*) data (Figure 2), it was possible to determine the optimal number of reference genes required for the relative quantification analysis and to investigate the effect of gene addition in this normalization. The data suggested that the addition to the two most stable genes (*α*TUB and GAPDH) considering a third gene (*V*_2/3_ = 0.15; Figure 2), a fourth (*V*_3/4_ = 0.14), or even more genes (*V*_4/5_ and *V*_5/6_; Figure 2) still exhibited desired values (below 0.15 as proposed by Vandesompele et al. [17]). To normalize the gene expression in the above mentioned sugarcane samples, *α*TUB, GAPDH, and H1 seem to be sufficient (Figure 1).

#### 3.2.2. NormFinder and BestKeeper Analysis

Basically, the gene expression stability ranking provided by the NormFinder and BestKeeper software exhibited the same order, with only the first two candidates switching places comparing the ranking (Table 3). The two software programs identified histone H1 (*M* = 0.28; CV ± SD = 1.06 ± 0.26) and *α*TUB (*M* = 0.32; CV ± SD = 1.06 ± 0.29) as the most stable genes, followed by GAPDH, 25S rRNA, UBQ, and SAMDC (see respective values in Table 3). Again, SAMDC showed the highest instability, in agreement with the GeNorm results. All the three software programs presented fairly consistent results showing the first three (H1, *α*TUB, and GAPDH) as the most stable and reliable genes for RT-qPCR data normalization. Two of them (H1 and *α*TUB) are reported as RT-qPCR normalizing genes suitable for sugarcane roots under abiotic stress for the first time.

### 3.3. Normalization of Target Genes

**Figure 3:**
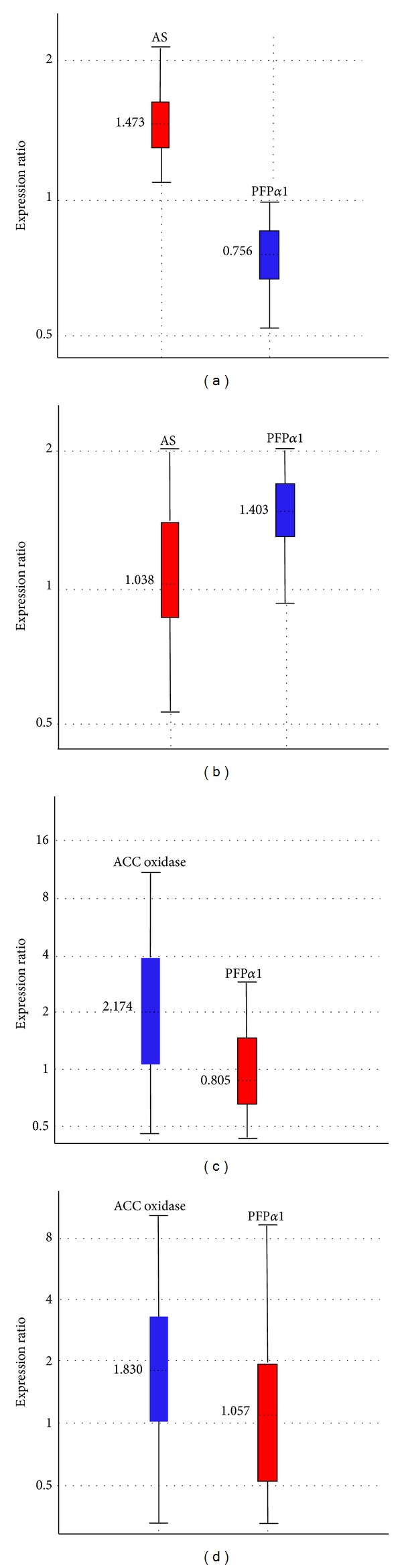
Relative expression of glutamine-dependent asparagine synthetase (AS), pyrophosphate-fructose 6-phosphate 1-phosphotransferase (PFP*α*1), plasma membrane intrinsic protein 1-1 (PIP1-1), and 1-aminocyclopropane-1-carboxylate oxidase (ACC oxidase) by the REST software v. 2.0.13 (after the ΔΔCq method) in cDNAs of sugarcane roots under abiotic stress (24 h of continuous dehydration or salt stress; 100 mM NaCl), normalized by the reference genes H1, *α*TUB, and GAPDH. (a) Tolerant bulk (CTC6, CTC15, SP83-2847, and SP83-5073 accessions) compared to its negative control. (b) Susceptible bulk (CTC9, CTC13, SP90-1638, and SP90-3414) compared to its negative control. (c) Salt-tolerant accession (RB931011) after 30 min of salt stress compared to its negative control. (d) RB931011 after 90 min of salt stress compared to its negative control. Relative expression with the median value (horizontal dotted line at the colored box) and range comprising 100% of the observations (horizontal bars), being 50% of them in the confidence interval at 95% (colored box).

In order to assess the applicability of the recommended histone H1, *α*TUB, and GAPDH as reference genes in relative expression studies using RT-qPCR, four targets based on sugarcane SuperSAGE unitags annotated as glutamine-dependent asparagine synthetase (AS, EC 6.3.5.4), pyrophosphate fructose-6-phosphate 1-phosphotransferase alpha subunit (PFP*α*1, EC 2.7.1.90), plasma membrane intrinsic protein (PIP1-1), and 1-aminocyclopropane-1-carboxylate oxidase (ACC oxidase, EC 1.14.17.4) were evaluated (Table 4). HT-SuperSAGE survey pointed SD282748 unitag as a potential AS being upregulated 1.92 times in the drought-tolerant bulk after the stress (24 h of continuous dehydration) as compared to the unstressed control while no relevant unitag frequency change was observed (*P* < 0.05) with the sensitive contrast (Table 4). The RT-qPCR relative quantification results confirmed the overexpressed status in relation to both cDNA bulks with 1.473-fold change for the tolerant bulk compared to its negative control (Figure 3(a)) and no significant change (1.038 times) considering the sensitive bulk in the comparative contrast (Figure 3(b)). In turn, SD179780 unitag (annotated as PFP*α*1) did not respond (*P* < 0.05) to water deficit stimulus in any contrast analyzed involving sugarcane drought-tolerant or -sensitive accessions, in agreement with the RT-qPCR results, showing constitutive expression of this gene (Figures 3(a) and 3(b), resp.). In an attempt to explore the use of the proposed reference genes, a target relative to the ASS122537 unitag (ACC oxidase) from the salt HT-SuperSAGE libraries, which was induced (UR) by the salt-tolerant accession (1.95 times after the salt stress exposition, NaCl 100 mM), showed overexpression after 30 min according to RT-qPCR results (Figure 3(c) and Table 4). Another target relative to the ASS140030 unitag (PIP1-1) from sugarcane after salt stress presented RT-qPCR results confirming the constitutive expression observed* in silico* (*P* < 0.05), even after the bulk has been opened in two times of salt exposition (30 and 90 min, Figures 3(c) and 3(d) and Table 4). The same PIP1-1 had been validated by RT-qPCR with cDNAs from the drought-tolerant and -sensitive sugarcane accessions (24 h of continuous dehydration) using GAPDH and 25S rRNA as the reference genes, in a previous work, showing differential expressions as expected by the SuperSAGE analysis [34].

## 4. Discussion

The understanding of sugarcane physiology under environmental stress remains under intensive research, due to the socioeconomic importance of this crop and the increasing unpredictability of environmental conditions worldwide. In this regard, gene expression analysis is an attractive approach to dissect plant physiological response to stress conditions. Nevertheless, reference gene selection has received limited attention in sugarcane. RT-qPCR is currently one of the most used techniques for gene expression analysis, due to its rapid, specific, and highly sensitive parameters. However, problems with RNA samples variations, standardization, and protocols efficiency (RNA extraction, RT, and qPCR) have routinely been observed [35]. Furthermore, the choice of normalizing genes remains one of the most time consuming and difficult steps in RT-qPCR. It requires reference genes to be constitutively expressed under external stimuli. Additionally, it needs to exhibit little or no behavior change in different cell types or tissues, as well as in specific developmental stages and experimental conditions [36–38]. Stal Papini-Terzi et al. [21] described transcriptional profile of signal transduction events in different sugarcane tissues, using reference genes selected based upon the literature (tubulin and actin), microarray data, and ESTs (polyubiquitin and 14-3-3 proteins). To individually normalize gene expression in sugarcane under certain conditions, Rocha et al. [22] relied upon four reference genes (14-3-3, polyubiquitin, GAPDH, and 25S rRNA). Moreover, Rodrigues et al. [23] used *β*-tubulin as the reference gene based on previous data [24]. To our knowledge, Iskandar et al. [24] represented an attempt to prospect stable sugarcane reference genes by checking the reliability of four genes (*β*-actin, *β*-tubulin, GAPDH, and 25S rRNA) in leaf, root, and internode tissues of some sugarcane cultivars and representatives of* Saccharum* genus, but none of them under abiotic stress. According to the authors, GAPDH was the most stable gene (CV = 51%) comparing different tissues, followed by *β*-actin and *β*-tubulin (CV = 81% and 68%, resp.); regarding species, *β*-actin showed the lowest coefficient of variation (31%) followed by GAPDH (33%). Although these methods are useful for prospecting candidate reference genes [21] or addressing gene expression using validated reference genes for target tissues [22, 23], selection using more appropriate statistical approaches should be the method of choice for identification of new reliable reference genes. In this way, software programs like GeNorm, NormFinder, and BestKeeper have assisted researchers by indicating reference genes suitable for expression profiling normalization studies [39]. In the present study, a group of potential reference genes (*α*TUB, GAPDH, H1, SAMDC, UBQ, and 25S rRNA) were evaluated by the three software programs, in order to evaluate their reliability for expression profiles normalization in sugarcane roots under abiotic stress (24 h of continuous dehydration). Basically, all three software programs pointed histone H1, *α*TUB, and GAPDH as the most reliable reference genes, with some of them switching places in the ranking. This set of genes was employed here as reference genes to validate sugarcane cDNAs relative to unitags from SuperSAGE libraries composed of roots of plants after stress exposition. Thus, the gene expression stability ranking provided by NormFinder and BestKeeper software showed the same order after the third place. Besides, based on the GeNorm analysis and* M-*values, all the six candidates may be suitable for normalizing gene expression data as presented here. But, combining the two best candidates (*α*TUB and H1) with the most variable one (SAMDC, *M* = 0.87), as reference genes normalizing the target AS (induced in SuperSAGE analysis), the REST software did not detect the expected overexpression (comparison 1, Table 5) due to the SAMDC largest standard deviation (SD) influencing the *P* value calculated, consequently leading to a false negative and possible misinterpretation of data. Alias, any other combination including SAMDC as reference gene did not reveal the alleged overexpression (comparisons 2–5, Table 5), indicating that this gene is not suitable for gene expression normalization in roots of sugarcane accessions under the evaluated stress. However, Hong et al. [40] reported SAMDC as the most reliable reference gene in grass* Brachypodium distachyon* when evaluated under four abiotic stress conditions (high salt (300 mM), cold (4°C for 5 h), heat (42°C for 2 h), and drought (400 mM mannitol)). In addition, Li and Chen [41], when describing SAMDC as a target gene, verified that this gene was induced in roots of rice seedlings at three leaf stages (after application of 171 mM salt for 24 h and 20 mM exogenous abscisic acid (ABA) and dehydration using 15% PEG6000). These results highlight the need to choose appropriate reference genes for each experiment, especially under stress conditions.

On the other hand, the other five genes (*α*TUB, GAPDH, H1, UBQ, and 25S rRNA) could be successfully employed in the normalization analyses, composing different combinations of reference genes (comparisons 6–11, Table 5), with similar results to that observed for the proposed set (H1, *α*TUB, and GAPDH). In relation to the 25S rRNA gene, it was the most abundant transcript (Cq 13.06), in agreement with results previously obtained from rice (Cq values of 15 [42]) and sugarcane (Cq values of 16.6 [24]). This can be explained by the fact that rRNA comprises the majority of total RNA present in a cell and, thus, further dilution for its use in RT-qPCR approaches [43] would be required. In the present work, only a 1 : 5 dilution was applied. Furthermore, the abundance of transcripts can affect the stability and, therefore, the normalizing results for the reference gene candidates [44]. In turn, GAPDH was also one of the most stable genes, confirming it to be an appropriate reference gene for experiments involving sugarcane roots under water deficit conditions. Concerning *α*TUB, it was indicated by GeNorm as the most stable gene using the bulks of accessions, both under regular irrigation and after 24 h of continuous dehydration. By the NormFinder and BestKeeper analysis, this gene was the second most appropriate reference gene. However, Fan et al. [38] assessing the reliability of reference genes in 14 different tissues and developmental stages of* Phyllostachys edulis *observed that *α*TUB showed a larger variation (*M* = 1.94) among all candidates. Similar results were reported by Zhong et al. [45] with litchi (*Litchi chinensis *Sonn.) under several experimental conditions (tissues, organs, developmental stages, and varieties), showing *α*TUB as the most variable gene among 10 candidates. Under biotic and abiotic stresses *α*TUB also displayed instability, as demonstrated by Die et al. [39], Hong et al. [40], and Zhu et al. [46]. Thus, the selection of suitable reference genes to normalize gene expression in sugarcane and other plant species seems to be essential because reference genes may be differently regulated in different species, displaying particular gene expression patterns [43]. The proposed reference genes (H1, *α*TUB, and GAPDH) validated the gene expression of sugarcane cDNAs related to SuperSAGE unitags, showing upregulation or even constitutive basis, in the mentioned drought assay. Among those targets, AS is a crucial component of the asparagine synthesis, acting as a key member in nitrogen assimilation, recycling, and storage in higher plants [47]. The overexpression results observed in the present study supported those found in a previous microarray [48] showing AS induction in wheat accession considered tolerant to drought after 36 h of irrigation suppression. AS were also detected by RT-PCR, using mRNA samples from roots and shoots (two-week-old plantlets), significantly induced after salinity (250 mM), osmotic stress (using mannitol 5.0% (w/v)), and exogenous abscisic acid (ABA) application (20 mM) [49]. Altogether, these results indicate the involvement of the AS gene in response to several stresses. Considering PFP*α*1, the constitutive expression observed in the present assay has been reported previously and was in agreement with Lim et al. [50] who demonstrated by semiquantitative RT-PCR that the expression of PFP*α*1 in wild-type* Arabidopsis* was also constitutive in different tissues (roots, leaves, and flowers) and also in distinct developmental stages (15, 25, and 45 days after planting). PFP*α*1 is responsible for the addition of phosphate to the second D-fructose 6-phosphate in the glycolysis pathway [51] and is essential for maintenance of carbohydrate metabolism and other processes in plant cells [50]. In sugarcane, PFPs are known to play a prominent role in sucrose accumulation, especially in immature and metabolically active tissues, taking part in glycolysis and in carbon compartmentalization [52].

The effort to explore the use of the proposed reference genes (H1, *α*TUB, and GAPDH) normalizing the expressions of sugarcane cDNAs (associated with SuperSAGE unitags) also from roots of plants under salt stress (100 mM) was effective in a preliminary study. This way, the induction of ASS122537 unitag (annotated as ACC oxidase, enzyme responsible to convert the ethylene precursor ACC to ethylene, [53]), as revealed by* in silico* SuperSAGE analysis, was confirmed by RT-qPCR after 30 minutes of salt exposition (tolerant accession), suggesting that rapid ethylene production is an adaptive response to the new conditions imposed by the salt stress to the evaluated genotype. Unfortunately this overexpression was not detected during the 90 min of stress exposition. Nevertheless, there is evidence that a variety of stressful conditions trigger the synthesis of ethylene [54]. Regarding salt stress, it has been observed that this hormone signaling may be required for triggering the tolerance process. Yang et al. [55], when evaluating* Arabidopsis* mutants (ein2-5, ein3-1, and ctr1-1) and wild plants ecotype Col-0, found that mutants insensitive to ethylene (ein2-5 or ein3-1) were more sensitive to saline stress when compared to their wild counterpart. The opposite was found in the mutant sensitive to ethylene (ctr1-1), which showed significant tolerance to salt stress. Concerning the PIP1-1, the RT-qPCR results showed no significant differences in any of the two sampled times, confirming the SuperSAGE results with the bulk comprising both sampling times. The same target in RT-qPCR assay confirmed differential expressions expected by the SuperSAGE analysis, with root cDNAs from the drought-tolerant and -sensitive sugarcane accessions (24 h of continuous dehydration) and GAPDH and 25S rRNA as the reference genes [34]. An explanation could be that the time intervals used for stress exposition were not enough for PIP1-1 expression. In rice, Guo et al. [56] reported PIP1-1 expression in response to salt stress (250 mM NaCl) after 2 h of stress exposition.

## 5. Conclusions

The potential of the six proposed reference genes (*α*TUB, GAPDH, H1, SAMDC, UBQ, and 25S rRNA) was confirmed after they were tested with cDNAs from sugarcane roots under drought stress (24 h of continuous dehydration) and analyzed by three different software programs (GeNorm, NormFinder, and BestKeeper). With the exception of SAMDC, all the other candidate genes seem to be suitable for sugarcane expression profiling normalization, but three of them (*α*TUB, H1, and GAPDH) were considered as the best reference genes. In this study, two new reference genes were reported for the first time for sugarcane (*α*TUB and H1), to undergo a RT-qPCR validation study involving expression in roots under abiotic stresses. Also, the present work pointed GAPDH and 25S rRNA genes, both indicated by Iskandar et al. [24], as reference genes in a previous study, also suitable for use with sugarcane root under abiotic stress. Using the proposed set of reference genes (*α*TUB, H1, and GAPDH), it was confirmed that the relative expression profile, with the aid of the REST software, of cDNAs was associated with unitags (26 bp) and annotated as AS and PFP*α*1, using a bulk of cDNAs relative to the drought-tolerant sugarcane accessions (four accessions, 24 h of continuous dehydration), in agreement with the HT-SuperSAGE data. Another two unitags (associated with ACC oxidase and PIP1-1) had their expression profiles validated by RT-qPCR, using cDNAs from sugarcane roots after salt stress exposition (100 mM NaCl), in an attempt to explore other possibilities using these reference genes. In conclusion, this set of reference genes will be useful for dissecting gene expression in sugarcane roots, especially in advanced transcriptomic studies using SuperSAGE or RNAseq approaches covering abiotic stresses.

## Supplementary Material

Supplementary Material showing the quality of the total RNAs from sugarcane roots under drought stress (FIGURE S1 - A), the amplicons of expected size for each gene used in the study, displaying primer specificity as required for RT-qPCR amplification (FIGURE S1 - B), and the melting curves showing a single peak for six potential reference genes (*α*TUB; GAPDH; H1; SAMDC; UBQ and 25S rRNA) and four targets (PFP*α*1; AS; PIP1-1 and ACC oxidase).
Supplementary Material (FIGURE S2) showing standard curves using a dilution series (100, 10^−1^, 10^−2^, 10^−3^ e 10^−4^) of six potential reference genes (*α*TUB; GAPDH; H1; SAMDC; UBQ and 25S rRNA) for sugarcane roots under drought stress and the respective curves for four targets (PFP*α*1; AS; PIP1-1 and ACC oxidase).
Supplementary Material (TABLE S2) based on MIQE checklist for authors, reviewers, and editors.
Supplementary Material (TABLE S1) showing Cq values of potential reference genes for gene expression normalization (RT-qPCR) with cDNAs from sugarcane roots under drought stress (24 h of continuous dehydration).

## Figures and Tables

**Table 1:** Potential reference genes and target genes with the individual accession numbers, annotations, and primer sequences.

Gene (accession numbers)	Source*	Predicted function	Description	Primer sequences (forward/reverse)
Reference genes				

*α*TUB (CN607271)	dbEST	Structural constituent of cytoskeleton	Alpha-tubulin	(F) CCATTGGCAAGGAGATTGTT (R) TCCACCAACTGCATTGAAGA

GAPDH (CA254672)	dbEST	Glycolysis, gluconeogenesis	Glyceraldehyde 3 phosphate dehydrogenase	(F) GGTTCACTTGAAGGGTGGTG (R) TGAGGTGTACCTGTCCTCGTT

H1 (CA116806)	[23]	Chromatin condensation	Histone H1	(F) CGCACACGCACACTGAAAG (R) CGGTGGCCATGATCAAAAA

SAMDC (CA127376)	[23]	Polyamine and triamine biosynthesis	S-Adenosylmethionine decarboxylase	(F) TGCTGCTGAAGACGCTGTTG (R) TCGCCTTCAAAGCAGTGTAGAAC

UBQ (CA077378)	dbEST	Protein degradation	Ubiquitin	(F) ACCGAAGGTTGCATTCAAGAC (R) GGGTTTGGGTCCGTTAGAAG

25S rRNA (BQ536525)	dbEST	Translation	25S ribosomal RNA	(F) GCAGCCAAGCGTTCATAG (R) CGGCACGGTCATCAGTAG

Target genes				

PFP*α*1 (XM_004973200.1)	dbEST	Carbohydrate metabolism, glycolysis	Pyrophosphate fructose-6-phosphate 1-phosphotransferase alpha subunit	(F) TTATGAGTTGCTGCGAGAGAAG (R) TATCTCAATGTCGCCCATGTAG

AS (FM212633.1)	dbEST	Asparagine synthesis	Glutamine-dependent asparagine synthetase	(F) CCAGAGAACACACCCACAAC (R) ATGCCACACTAGGACCTCCA

PIP1-1 (CF572112)	dbEST	Integral to membrane, water transport	Plasma membrane intrinsic protein 1-1	(F) GTTCCTATCCTTGCCCCACT (R) AGGCGTGATCCCTGTTGTAG

ACC oxidase (TC127289)	SoGI	Ethylene biosynthesis	1-Aminocyclopropane-1-carboxylate oxidase	(F) GGGACCTCTTGCAGATAATGTC (R) CTCTGGCAATGGTCCATAGAA

*Databases: dbEST (NCBI; http://www.ncbi.nlm.nih.gov/) and Gene Index (SoGI; http://compbio.dfci.harvard.edu/tgi/); Rodrigues et al. [23]. *α*TUB: alpha-tubulin; GAPDH: glyceraldehyde 3 phosphate dehydrogenase; H1: histone H1; SAMDC: S-adenosylmethionine decarboxylase; UBQ: ubiquitin; 25S rRNA: 25S ribosomal RNA; PFP*α*1: pyrophosphate fructose-6-phosphate 1-phosphotransferase alpha subunit (EC 2.7.1.90); AS: glutamine-dependent asparagine synthetase (EC 6.3.5.4); PIP1-1: plasma membrane intrinsic protein; ACC oxidase: 1-aminocyclopropane-1-carboxylate oxidase (EC 1.14.17.4).

**Table 2:** Parameters derived from RT-qPCR data analysis*, including PCR amplification efficiency (*E*) established by calibration curves for each quantified target.

Gene	Tm (°C)	Product size (bp)	Average Cq	*E* (%)	NTC (Cq)	Correlation coefficient (*R*)	Slope	*Y* intercept
*α*TUB	75.9	104	27.48	99.53	35.82	0.998	−3.33	37.34
GAPDH	81.8	100	23.69	100.89	N.D.	0.984	−3.30	41.40
H1	78.0	57	24.76	97.41	33.59	0.999	−3.39	37.92
SAMDC	79.0	60	22.74	99.66	36.62	0.992	−3.33	34.36
UBQ	81.2	153	24.64	98.34	N.D.	0.999	−3.36	37.86
25S rRNA	82.9	108	14.00	99.82	N.D.	0.999	−3.33	35.54
PFP*α*1	83.6	151	21.92	92.99	32.90	0.988	−3.50	40.44
AS	79.8	112	24.94	105.05	N.D.	0.990	−3.21	40.26
PIP1-1	84.6	134	24.47	95.55	N.D.	0.995	−3.43	37.50
ACC oxidase	82.2	152	30.21	93.52	N.D.	0.990	−3.49	44.65

*Based on MIQE Guidelines [26]. Tm: melting temperature (°C); N.D.: not detected; NTC: no template control; *α*TUB: alpha-tubulin; GAPDH: glyceraldehyde 3 phosphate dehydrogenase; H1: histone H1; UBQ: ubiquitin; SAMDC: S-adenosylmethionine decarboxylase; 25S rRNA: 25S ribosomal RNA; PFP*α*1: pyrophosphate fructose-6-phosphate 1-phosphotransferase alpha subunit (EC 2.7.1.90); AS: glutamine-dependent asparagine synthetase (EC 6.3.5.4); PIP1-1: plasma membrane intrinsic protein; ACC oxidase: 1-aminocyclopropane-1-carboxylate oxidase (EC 1.14.17.4).

**Table 3:** Expression stability values for sugarcane candidate calculated by NormFinder and BestKeeper software.

Ranking	NormFinder analysis		BestKeeper analysis	
	Gene name	*M*	Gene name	CV ± SD
1	*α*TUB	0.11	H1	(1.06 ± 0.26)
2	H1	0.16	*α*TUB	(1.06 ± 0.29)
3	GAPDH	0.19	GAPDH	(1.33 ± 0.31)
4	25S rRNA	0.28	25S rRNA	(3.89 ± 0.55)
5	UBQ	0.31	UBQ	(2.04 ± 0.50)
6	SAMDC	0.33	SAMDC	(2.53 ± 0.57)

*M*: average expression stability value; CV: coefficient of variance; SD: standard deviation; *α*TUB: alpha-tubulin; H1: histone H1; GAPDH: glyceraldehyde 3 phosphate dehydrogenase; 25S rRNA: 25S ribosomal RNA; UBQ: ubiquitin; SAMDC: S-adenosylmethionine decarboxylase.

**Table 4:** Relative expression rates of target genes (PFP*α*1, AS, PIP1-1, and ACC oxidase) based on RT-qPCR with roots, cDNAs of sugarcane accessions under abiotic stress, and respective unitag regulation by SuperSAGE analysis covering drought^a^ stress (24 h of continuous dehydration) or salt^b^ stress (100 mM NaCl).

Unitag	Annotation	SuperSAGE [FC/Regulation*]		RT-qPCR^∗&^	
		Tolerant	Sensitive	Tolerant	Sensitive
SD282748^a^	AS	1.92^#^/UR	−1.10^#^/ns	1.473^#^/UR	1.038^#^/ns
D179780^a^	PFP*α*1	1.99^#^/ns	−1.07^#^/ns	0.756^#^/ns	1.403^#^/ns
ASS122537^b^	ACC oxidase	1.95/UR	—	2.174/UR (30′) 1.830/ns (90′)	—
ASS140030^b^	PIP1-1	−1.31/ns	—	0.805/ns (30′) 1.057/ns (90′)	—

AS: glutamine-dependent asparagine synthetase (EC 6.3.5.4); PFP*α*1: pyrophosphate fructose-6-phosphate 1-phosphotransferase alpha subunit (EC 2.7.1.90); ACC oxidase: 1-aminocyclopropane-1-carboxylate oxidase (EC 1.14.17.4); PIP1-1: plasma membrane intrinsic protein. ^#^Bulk with four accessions by each library; FC: fold change [ratio of the frequencies (normalized to 1,000,000) observed in the stressed library in relation to the control library]; ^&^relative expression level by REST software (v.2.0.13) after the ΔΔCq method, *P < 0.05 [27]; UR: upregulated; ns: not significant at *P* < 0.05. The time in the parentesis represents the salt stress exposition (min).

**Table 5:** Potential reference gene combinations (and number of genes involved in each comparison) used in gene expression normalization of glutamine-dependent asparagine synthetase (AS), with cDNAs of sugarcane accessions (root under drought stress, 24 h of continuous dehydration).

Comparison	Gene combinations	Number of genes	Expression* value	*P* value	Regulation
1	*α*TUB, H1, and SAMDC	3	1.431	0.112	ns
2	GAPDH, 25S rRNA, and SAMDC	3	1.482	0.009	ns
3	*α*TUB, H1, GAPDH, 25S rRNA, and SAMDC	5	1.496	0.113	ns
4	*α*TUB, H1, UBQ, and SAMDC	4	1.557	0.105	ns
5	UBQ and SAMDC	2	1.595	0.305	ns
6	*α*TUB and H1	2	1.519	0.000	UR
7	GAPDH and 25S rRNA	2	1.600	0.017	UR
8	*α*TUB, H1, GAPDH, and 25S rRNA	4	1.559	0.011	UR
9	*α*TUB, H1, and UBQ	3	1.667	0.033	UR
10	GAPDH, 25S rRNA, and UBQ	3	1.725	0.017	UR
11	*α*TUB, H1, GAPDH, 25S rRNA, and UBQ	5	1.640	0.017	UR

*REST software analysis after the ΔΔCq method. UR: upregulated; ns: not significant at *P* < 0.05; *α*TUB: alpha-tubulin; H1: histone H1; SAMDC: S-adenosylmethionine decarboxylase; GAPDH: glyceraldehyde 3 phosphate dehydrogenase; 25S rRNA: 25S ribosomal RNA; UBQ: ubiquitin.
