# Supplementary material for: Validation of Novel Reference Genes for Reverse Transcription Quantitative Real-Time PCR in Drought-Stressed Sugarcane
Source: ScientificWorldJournal. 2014 Jun 2;2014:357052. doi: 10.1155/2014/357052 (PMC4060590; doi:10.1155/2014/357052)
Supplement: Supplementary file 1 — Supplementary Material showing the quality of the total RNAs from sugarcane roots under drought stress (FIGURE S1 - A), the amplicons of expected size for each gene used in the study, displaying primer specificity as required for RT-qPCR amplification (FIGURE S1 - B), and the melting curves showing a single peak for six potential reference genes (αTUB; GAPDH; H1; SAMDC; UBQ and 25S rRNA) and four targets (PFPα1; AS; PIP1-1 and ACC oxidase). Supplementary Material (FIGURE S2) showing standard curves using a dilution series (100, 10−1, 10−2, 10−3 e 10−4) of six potential reference genes (αTUB; GAPDH; H1; SAMDC; UBQ and 25S rRNA) for sugarcane roots under drought stress and the respective curves for four targets (PFPα1; AS; PIP1-1 and ACC oxidase). Supplementary Material (TABLE S2) based on MIQE checklist for authors, reviewers, and editors. Supplementary Material (TABLE S1) showing Cq values of potential reference genes for gene expression normalization (RT-qPCR) with cDNAs from sugarcane roots under drought stress (24 h of continuous dehydration). [file 357052.f1.pdf]

Supplementary Materials

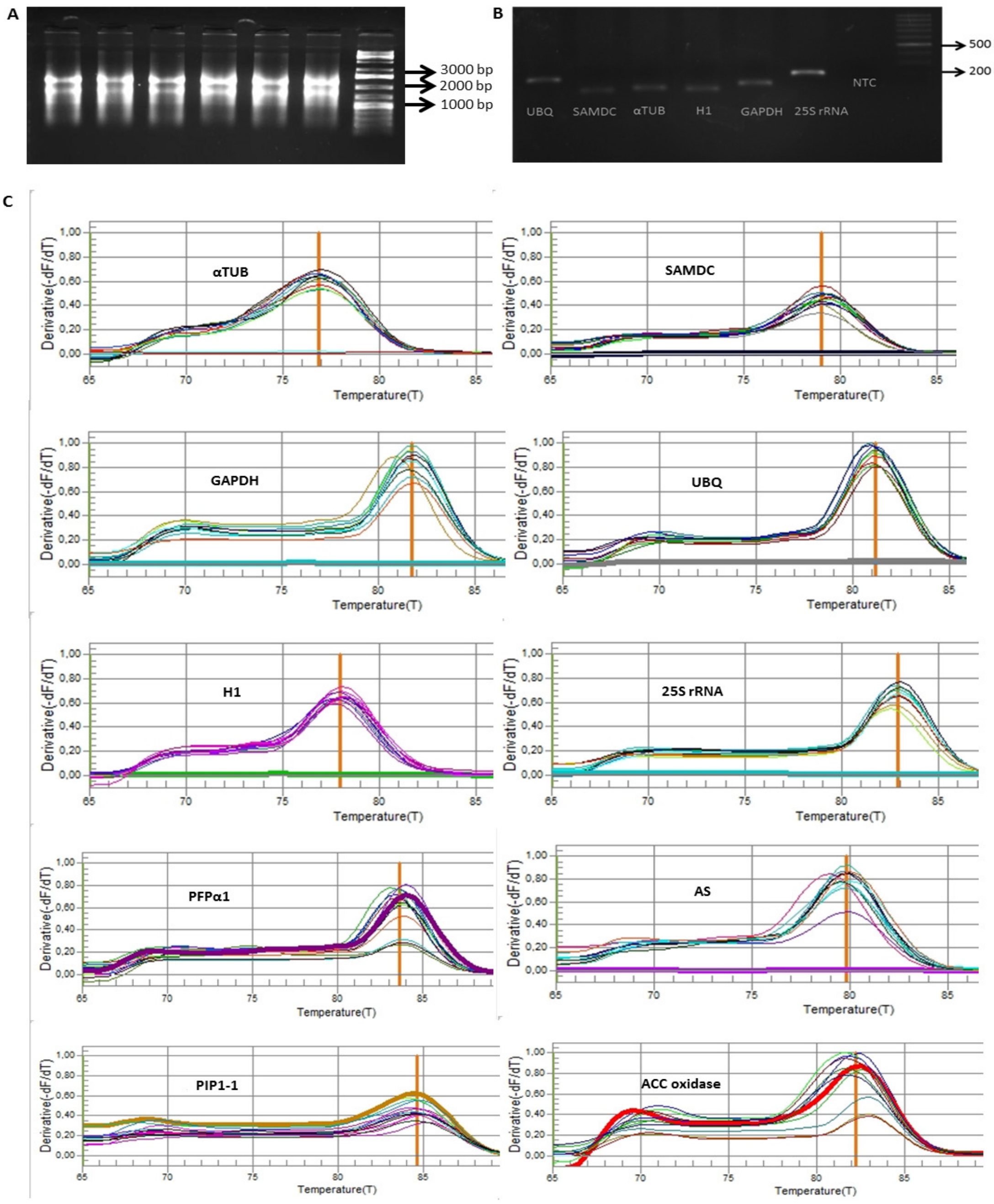

FIGURE S1: (A) Total RNA from sugarcane roots under drought stress treated with DNAse. Thermo Scientific GeneRuler ™ 1kb Ladder. (B) Agarose gel (1.5 %) showing the specific products of expected size for each gene, displaying primer specificity as required for RT-qPCR amplification. Thermo Scientific GeneRuler™ 100 pb DNA Ladder.(C) Melting curves showing a single peak for the candidate genes (αTUB: Alpha-tubulin; GAPDH: Glyceraldehyde 3 phosphate dehydrogenase; H1: Histone H1; SAMDC: S-adenosylmethionine decarboxylase; UBQ: Ubiquitin; 25S rRNA: 25S ribosomal RNA) and targets (PFPα1: Pyrophosphate fructose-6-phosphate 1-phosphotransferase alpha subunit; AS: Glutamine-dependent asparagine synthetase; PIP1-1: Plasma membrane intrinsic protein 1-1; ACC oxidase: 1-aminocyclopropane-1-carboxylate oxidase).

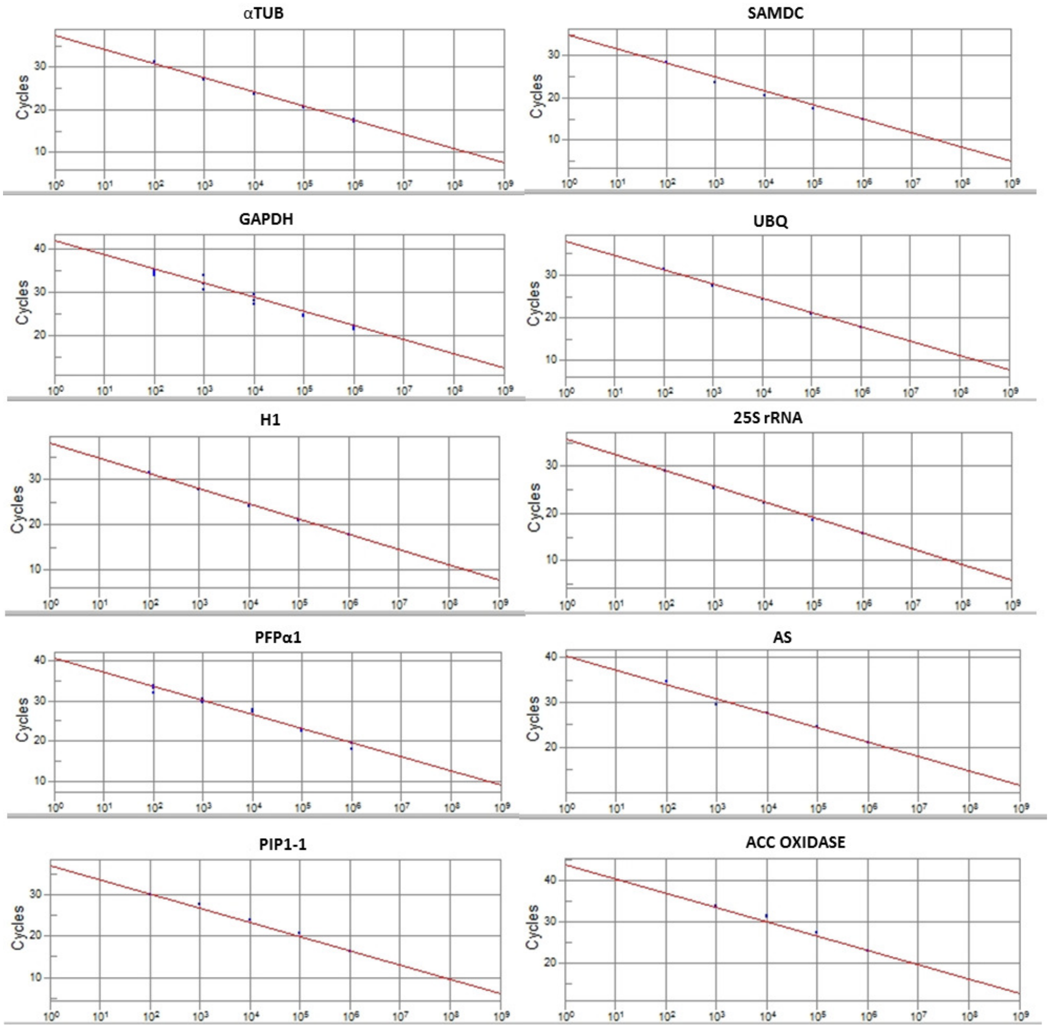

FIGURE S2: Standard curves using a dilution series (1, 10^{-1}, 10^{-2}, 10^{-3} e 10^{-4}) of potential reference genes for sugarcane roots under drought stress (αTUB: Alpha-tubulin; GAPDH: Glyceraldehyde 3 phosphate dehydrogenase; H1: Histone H1; SAMDC: S-adenosylmethionine decarboxylase; UBQ: Ubiquitin; 25S rRNA: 25S ribosomal RNA) and the respective curves for targets (PFPα1: Pyrophosphate fructose-6-phosphate 1-phosphotransferase alpha subunit; AS: Glutamine-dependent asparagine synthetase; PIP1-1: Plasma membrane intrinsic protein 1-1; ACC oxidase: 1-aminocyclopropane-1-carboxylate oxidase).

TABLE S1. Cq values of potential reference genes for gene expression normalization (RT-qPCR) with cDNAs from sugarcane roots under drought stress (24 h of continuous dehydration).

Sample*	αTUB	GAPDH	H1	SAMDC	UBQ	25S rRNA
1	27.18	23.89	25.56	22.43	23.70	14.85
2	28.00	23.51	24.42	22.00	24.55	14.46
3	27.96	23.97	24.67	23.22	25.66	13.88
4	27.47	23.48	24.94	22.03	24.21	14.29
5	27.15	23.78	24.48	22.05	25.11	14.61
6	27.38	23.10	25.14	22.17	25.19	14.53
7	27.41	23.91	24.38	23.47	24.07	13.43
8	27.19	24.23	24.55	24.24	24.52	13.21
9	27.71	24.23	24.97	23.49	23.74	13.68
10	27.08	23.29	24.54	22.47	24.88	13.06
11	27.23	23.47	24.71	22.62	24.88	13.48
12	28.00	23.39	24.77	22.73	25.11	14.54

*1-3: bulk of tolerant genotypes without stress (control); 4-6: bulk of tolerant genotypes under stress; 7-9: bulk of sensitive genotypes without stress (control); 10-12: bulk of sensitive genotypes under stress. αTUB: Alpha-tubulin; GAPDH: Glyceraldehyde 3 phosphate dehydrogenase; H1: Histone H1; SAMDC: S-adenosylmethionine decarboxylase; UBQ: Ubiquitin; 25S rRNA: 25S ribosomal RNA.

TABLE S2: MIQE checklist for authors, reviewers, and editors (see reference [32])[1].

	IMPORTANCE	Yes/No/NA[*]
**Experimental design**		
Definition of experimental and control groups	E	Yes
Number within each group	E	Yes
Assay carried out by the core or investigator's laboratory?	D	Yes
Acknowledgment of authors' contributions	D	Yes
**Sample**		
Description	E	Yes
Volume/mass of sample processed	D	Yes
Microdissection or macrodissection	E	NA
Processing procedure	E	Yes
If frozen, how and how quickly?	E	Yes
If fixed, with what and how quickly?	E	NA
Sample storage conditions and duration (especially for FFPE[2] samples)	E	Yes
**Nucleic acid extraction**		
Procedure and/or instrumentation	E	Yes
Name of kit and details of any modifications	E	Yes
Source of additional reagents used	D	Yes
Details of DNase or RNase treatment	E	Yes
Contamination assessment (DNA or RNA)	E	Yes
Nucleic acid quantification	E	Yes
Instrument and method	E	Yes
Purity (A260/A280)	D	Yes
Yield	D	No
RNA integrity: method/instrument	E	Yes
RIN/RQI or Cq of 3_ and 5_ transcripts	E	NA
Electrophoresis traces	D	NA
Inhibition testing (Cq dilutions, spike, or other)	E	Yes
**Reverse transcription**		
Complete reaction conditions	E	Yes
Amount of RNA and reaction volume	E	Yes
Priming oligonucleotide (if using GSP) and concentration	E	NA
Reverse transcriptase and concentration	E	Yes
Temperature and time	E	Yes
Manufacturer of reagents and catalogue numbers	D	Yes
Cqs with and without reverse transcription	D[3]	No
Storage conditions of cDNA	D	Yes
**qPCR target information**		
Gene symbol	E	Yes
Sequence accession number	E	Yes
Location of amplicon	D	Yes
Amplicon length	E	Yes
In silico specificity screen (BLAST, and so on)	E	Yes
Pseudogenes, retropseudogenes, or other homologs?	D	NA
Sequence alignment	D	No
Secondary structure analysis of amplicon	D	No
Location of each primer by exon or intron (if applicable)	E	Yes
What splice variants are targeted?	E	NA
**qPCR oligonucleotides**		
Primer sequences	E	Yes
RTPrimerDB identification number	D	No
Probe sequences	D[4]	NA
Location and identity of any modifications	E	No
Manufacturer of oligonucleotides	D	Yes
Purification method	D	Yes

Table S2. *Cont.*

**qPCR protocol**		
Complete reaction conditions	E	Yes
Reaction volume and amount of cDNA/DNA	E	Yes
Primer, (probe), $Mg2^{+}$, and dNTP concentrations	E	Yes
Polymerase identity and concentration	E	Yes
Buffer/kit identity and manufacturer	E	Yes
Exact chemical composition of the buffer	D	Yes
Additives (SYBR Green I, DMSO, and so forth)	E	Yes
Manufacturer of plates/tubes and catalog number	D	Yes
Complete thermocycling parameters	E	Yes
Reaction setup (manual/robotic)	D	Yes
Manufacturer of qPCR instrument	E	Yes
**qPCR validation**		
Evidence of optimization (from gradients)	D	No
Specificity (gel, sequence, melt, or digest)	E	Yes
For SYBR Green I, Cq of the NTC	E	Yes
Calibration curves with slope and y intercept	E	Yes
PCR efficiency calculated from slope	E	Yes
CIs for PCR efficiency or SE	D	No
r2 of calibration curve	E	Yes
Linear dynamic range	E	NA
Cq variation at LOD	E	NA
CIs throughout range	D	NA
Evidence for LOD	E	NA
If multiplex, efficiency and LOD of each assay	E	NA
**Data analysis**		
qPCR analysis program (source, version)	E	Yes
Method of Cq determination	E	Yes
Outlier identification and disposition	E	Yes
Results for NTCs	E	Yes
Justification of number and choice of reference genes	E	Yes
Description of normalization method	E	Yes
Number and concordance of biological replicates	D	Yes
Number and stage (reverse transcription or qPCR) of technical replicates	E	Yes
Repeatability (intraassay variation)	E	Yes
Reproducibility (interassay variation, CV)	D	Yes
Power analysis	D	Yes
Statistical methods for results significance	E	Yes
Software (source, version)	E	Yes
Cq or raw data submission with RDML	D	No

[*]Not Applicable; [1]All essential information (E) must be submitted with the manuscript. Desirable information (D) should be submitted if available. If primers are from RTPrimerDB, information on qPCR target, oligonucleotides, protocols, and validation is available from that source; [2]FFPE, formalin-fixed, paraffin-embedded; RIN, RNA integrity number; RQI, RNA quality indicator; GSP, gene-specific priming; dNTP, deoxynucleoside triphosphate; [3]Assessing the absence of DNA with a no–reverse transcription assay is essential when first extracting RNA. Once the sample has been validated as DNA free, inclusion of a no–reverse transcription control is desirable but no longer essential; [4]Disclosure of the probe sequence is highly desirable and strongly encouraged; however, because not all vendors of commercial predesigned assays provide this information, it cannot be an essential requirement. Use of such assays is discouraged.
